# Significant Performance Enhancement in Asymmetric Supercapacitors based on Metal Oxides, Carbon nanotubes and Neutral Aqueous Electrolyte

**DOI:** 10.1038/srep15551

**Published:** 2015-10-23

**Authors:** Arvinder Singh, Amreesh Chandra

**Affiliations:** 1 Department of Physics, Indian Institute of Technology Kharagpur, Kharagpur-721302, West Bengal, India

## Abstract

Amongst the materials being investigated for supercapacitor electrodes, carbon based materials are most investigated. However, pure carbon materials suffer from inherent physical processes which limit the maximum specific energy and power that can be achieved in an energy storage device. Therefore, use of carbon-based composites with suitable nano-materials is attaining prominence. The synergistic effect between the pseudocapacitive nanomaterials (high specific energy) and carbon (high specific power) is expected to deliver the desired improvements. We report the fabrication of high capacitance asymmetric supercapacitor based on electrodes of composites of SnO_2_ and V_2_O_5_ with multiwall carbon nanotubes and neutral 0.5 M Li_2_SO_4_ aqueous electrolyte. The advantages of the fabricated asymmetric supercapacitors are compared with the results published in the literature. The widened operating voltage window is due to the higher over-potential of electrolyte decomposition and a large difference in the work functions of the used metal oxides. The charge balanced device returns the specific capacitance of ~198 F g^−1^ with corresponding specific energy of ~89 Wh kg^−1^ at 1 A g^−1^. The proposed composite systems have shown great potential in fabricating high performance supercapacitors.

There is a growing demand to bring a step change in the specific power and energy delivered by supercapacitors. Such increase, along with the intrinsic advantage of fast charging/discharging rates, long cycle life (>10,000 cycles), and wide operational temperature range will allow the supercapacitors to compete with Li-ion batteries^1^. High performance supercapacitors have the capability to become an integral component of hybrid electric vehicles, back-up power supplies, mobiles, laptops, video cameras, signal transmitters, wearable electronics, etc.^2^. The specific energy (E) of supercapacitors can be enhanced by increasing the operating voltage window (V) and/or capacitance (C) as E = ½CV^2^. Consequently, supercapacitors employing ionic liquids or acetonitrile based electrolytes with operational voltage in the range 3-4 V have been reported^3^,^4^. However, the limited specific power and toxicity of these supercapacitors are the major limiting factors^5^,^6^. Aqueous electrolytes can be used as an alternative to their non-aqueous counterparts with an issue of narrow operating voltage window (~1.2 V), which mostly obstructs the specific energy of SCs^7^,^8^. Therefore, the fabrication of asymmetric supercapacitors (ASCs) in aqueous electrolytes is attaining more prominence to expand the operating voltage window. In ASCs, the implementation of the two appropriate electrode materials in the same electrolyte can add together their overlapped/non-overlapped operating voltage window. Therefore, an asymmetric cell configuration with correctly charge-balanced electrodes endows ASCs with the advantage of an extended cell voltage and high specific energy^9^.

Supercapacitors with reasonably high specific capacitance values are being fabricated using nano-sized transition metal oxides (e.g., MnO_2_, SnO_2_, Fe_3_O_4_, MoO_3_, V_2_O_5_ etc.)^10^,^11^,^12^,^13^,^14^,^15^,^16^. However, the low electrical conductivity of these metal oxides undermines their cyclic stability and limits the specific power that can be extracted. Consequently, use of composites with conventional carbon based materials having high electrical conductivity, surface area, chemical and mechanical stability is being^17^,^18^,^19^. The specific energy and coulombic efficiencies of such supercapacitors are predominantly decided by the characteristics of the electroactive material (EAM) taking part in the redox processes. The loss of performance is directly linked to the low capacitive negative electrode materials^20^,^21^.

Presently, amongst the various oxides being investigated as electrode material, tin (IV) oxide is cost-effective with good electrochemical response and easy synthesis^22^,^23^. Similarly, nanostructures of vanadium (V, IV) oxide are explored as pseudocapacitive materials due to their high physical and chemical stability^24^,^25^. In this paper, it is shown that the synergistic effect of MWCNTs with electroactive materials SnO_2_ and V_2_O_5_ can lead to appreciable increase in the specific capacitance. The charge balanced device assembled in 0.5 M Li_2_SO_4_ can be operated up to 1.8 V with no signature of gaseous evolution at upper bound of the potential. This allows the device to reach the maximum specific capacitance of ~198 F g^−1^ and specific energy of ~89 Wh kg^−1^. The fabricated ASCs also show good rate capability and retain ~96% of their initial specific capacitance value, even after 1200 cycles at 2 A g^−1^ charging/discharging current. The reasons contributing to the enhancement of specific energy are explained using the relevant theoretical models.

## Results

### Physical characterizations

**Figure 1:**
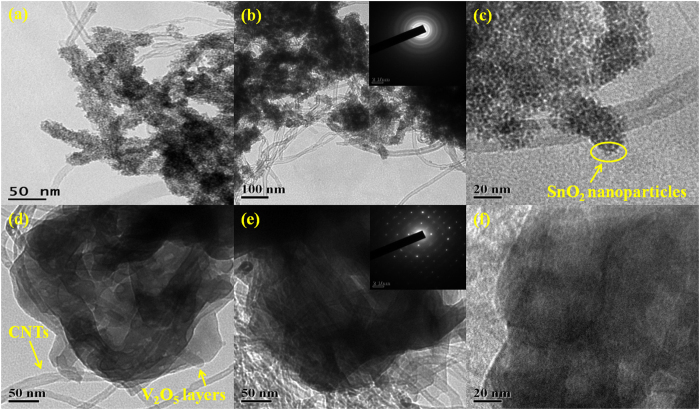
TEM micrographs for (a–c) MWS; (d–f) MWV composite.

Figure 1a–c shows the high resolution transmission electron microscopy (TEM) micrographs of the composite systems. From the TEM micrographs, it can be seen that spherical SnO_2_ nanoparticles and layered V_2_O_5_ are uniformly dispersed in the MWCNTs (MW) matrix. The elemental mapping shown in supplementary Fig. S1 confirms this inference. Supplementary Fig. S2 depicts FESEM micrographs for MWCNTs/SnO_2_ (MWS) and MWCNTs/V_2_O_5_ (MWV) whereas supplementary Fig. S3 shows the FESEM and TEM micrographs of spherical SnO_2_ nanoparticles and V_2_O_5_. It is important to mention that the phase formation, chemical state, and nature of bonding associated with the metal oxide were determined using the standard analysis of X-ray diffraction (Supplementary Fig. S4), X-ray photoelectron spectroscopy (XPS) (Supplementary Fig. S5), Fourier transform infrared spectroscopy (FTIR) and thermogravimetric analysis (TGA) (Supplementary Fig. S6) data. The details are given in the supplementary data.

**Figure 2:**
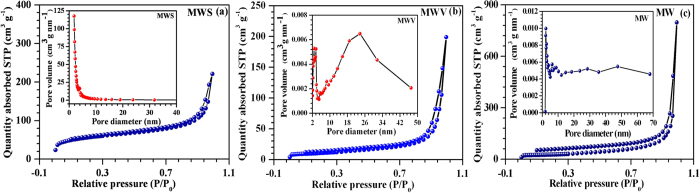
BET adsorption-desorption isotherms and pore size distribution for (a) MWS; (b) MWV; (c) MW sample.

**Figure 3:**
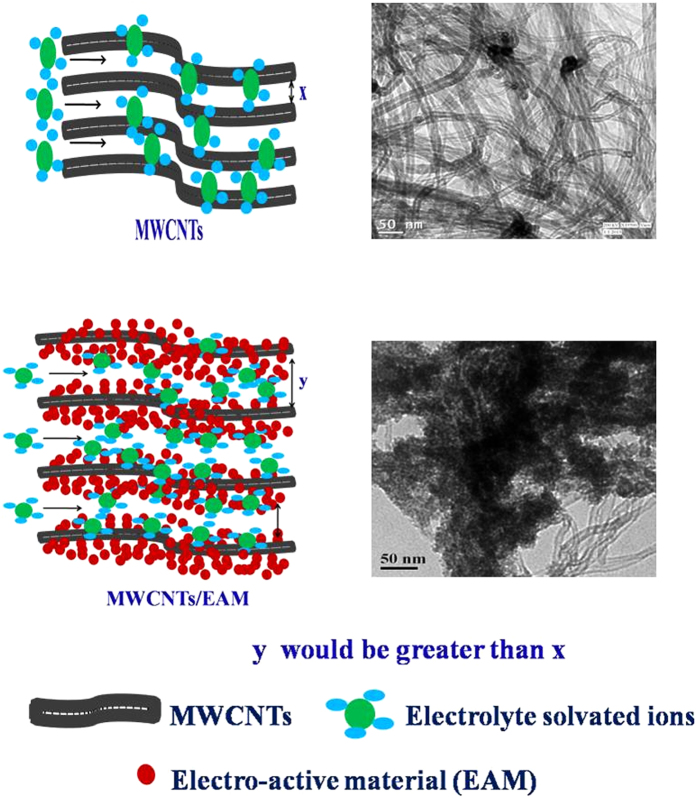
Theoretically conceptualized model to explain the opening of entangled carbon nanotubes as a result of composite formation.

The adsorption and desorption isotherms observed for the MWS composite was similar to IV-type isotherm with H3 hysteresis suggesting meso-porosity with capillary pore structures (see Fig. 2a). Using the data, it could be inferred that the majority of the pores had dimensions <10 nm. Figure 2b,c shows similar isotherms obtained in MWV and MWCNTs (MW) samples, respectively. The Brunauer–Emmett–Teller (BET) surface areas for the MWS, MWV and MW samples were found to be ~187 m^2^ g^−1^, 37 m^2^ g^−1^ and 92 m^2^ g^−1^, respectively. The MWV composite exhibited pores located in the mesopores range ~2.7 to 23.6 nm. The formation of such mesoporous structure can be attributed to the opening of the entangled CNTs due to the presence of metal oxides and voids formed during the packing of the different crystallites. This is schematically shown in Fig. 3. Meso-porosity is a desirable attribute for supercapacitor electrode assemblies as it can provide increased number of channels for the diffusion of electrolyte ions leading to enhanced charge storage.

### Individual electrochemical performance and charge-balancing

**Figure 4:**
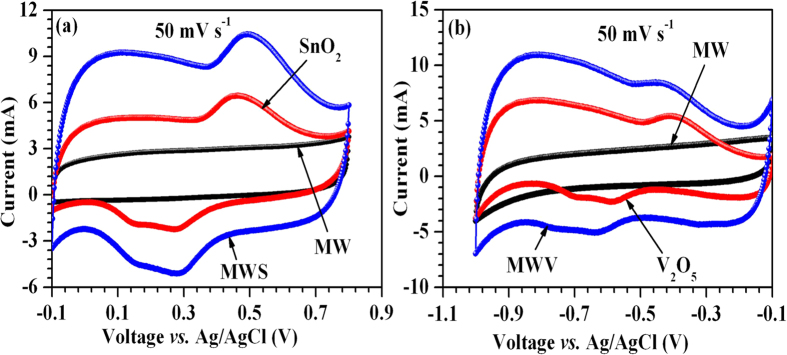
Synergistic effect between MWCNTs and (a) SnO_2_; (b) V_2_O_5_.

For sustainable device operation, ensuring charge balance condition is essential. Therefore, individual electrochemical performances for all the active materials were investigated using a three-electrode system comprising a saturated KCl Ag/AgCl reference electrode and a Pt counter electrode. Figure 4a depicts the CVs for the MW, SnO_2_ and MWS composite collected in the positive potential range at 50 mV s^−1^. A rectangular shaped CV was observed for the MW sample while redox peaks were discernible with SnO_2_ and MWS composite samples. The CV loop for MWS exhibited larger area (specific capacity ~218 F g^−1^) in comparison to that observed for MW or SnO_2_ nanoparticles alone (~29 F g^−1^ and ~136 F g^−1^). This can be explained by knowing the fact that MWS had higher surface area, enhanced porosity and improved conductivity, that would lead to effective utilization of the inner bulk i.e., positive synergistic effect. The CV curves recorded in the negative potential range for MW, V_2_O_5_ and MWV composite are shown in Fig. 4b. MWV also showed superior charge storage capacity (242 F g^−1^) in comparison to only MW or V_2_O_5_ (64 and 127 F g^−1^, respectively).

**Figure 5:**
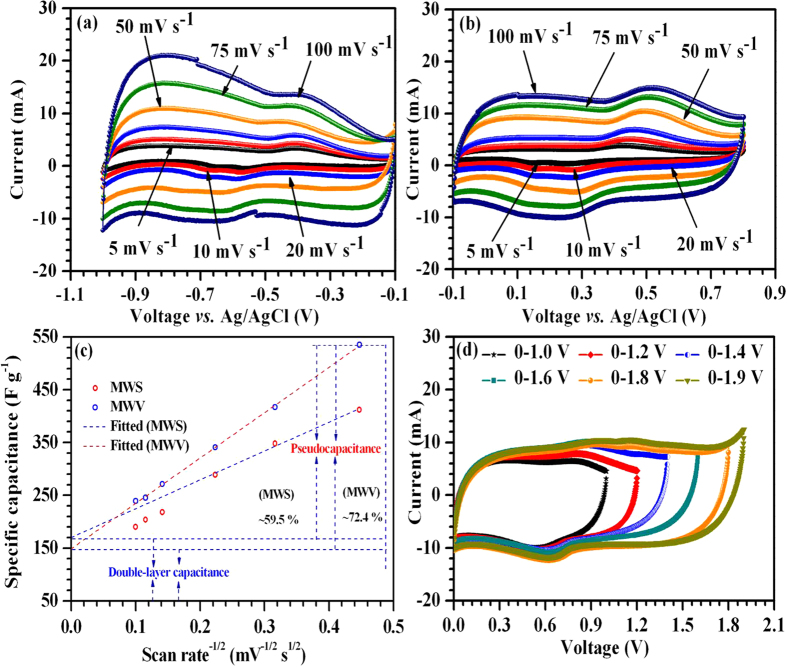
(**a,b**) Three electrode CV curves MWS and MWV, respectively; (**c**) quantification of double-layer and pseudocapacitance; (**d**) explaining stable voltage window for ASCs.

Figure 5a shows the CV curves of MWS collected at different scan rates between −0.1 to 0.8 V. Nearly rectangular shaped CV curves with pronounced redox peaks were observed. These are linked to the intercalation/de-intercalation of electrolyte ions into the SnO_2_ matrix (SnO_2_ + M^+^+ e^−^ ↔ SnOOM where M^+^ is the proton or electrolyte cation) and/or the redox reactions of the various functional groups present on the surface of MWCNTs^26^,^27^,^28^. In the composite material, MWCNTs provide the conducting pathways for the electron transfer while the SnO_2_ nanoparticles control the redox reactions with the electrolyte ions. The uncovered surface of MWCNTs can also exhibit double layer capacitance due to the adsorption of the electrolyte ions. As a consequence of positive synergistic effect, a maximum specific capacitance of ~412 F g^−1^ could be achieved using MWS composite material at a scan rate of 5 mV s^−1^. Equation (1) was used to calculate the specific capacitance (*C*):


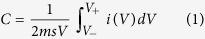


where *m* is the mass of the active material excluding mass of the binder (with mass of positive electrode m_+_ = 1 mg), *s* is the scan rate, *V_−_* and *V*_*+*_ represent the lower and upper voltage value of the voltage window range *V*, and *i(V)* denotes the corresponding current response.

The specific capacitance values at 10, 20, 50, 75 and 100 mV s^−1^ were found to be ~348, 289, 218, 204 and 190 F g^−1^, respectively. This shows good rate capability of MWS composite material with ~54% capacitance fade at a scan rate of 100 mV s^−1^. The CVs at various scan rates for the MWV composite in the negative potential range (−0.1 to −1.0 V) was also recorded and is shown in Fig. 5b. Similar to the earlier case, nearly horizontal quasi-rectangular CVs were observed with sharp redox peaks. This can be attributed to the insertion/de-insertion of electrolyte cations into V_2_O_5_/VO_2_ layered structures and/or redox reactions associated with surface functionality of the MWCNTs^29^,^30^,^31^. The positive synergistic effect between vanadium oxide and MW leads to a maximum specific capacitance of ~535 F g^−1^ at 5 mV s^−1^ scan rate.

The specific capacitance for MWV and MWS will be the sum of double layer (adsorption process) and pseudocapacitance (due to redox reaction, intercalation and diffusion inside the bulk active material). The variation of cumulated specific capacitance is shown in Fig. 5c. This is found to decrease linearly at a rate proportional to *v*^−1/2^ (*v* is the scan rate). The deviation from the linearity at higher scan rates appears due to the reduced utilization of the active material. Therefore, these data points were excluded during the linear fitting. The extrapolation of fitted line towards *v*^−1/2^ → 0 (infinite scan rate) gives the capacitance that is expected to originate electrostatically (adsorption) near the surface due to the slow pseudocapacitive processes^32^. The contribution of the electrode surface in the MWS (40.5%) is relatively high in comparison to MWV (27.6%). This can be attributed to relatively high surface area of the MWS composite which would result in a large interfacial contact region with the electrolyte.

The optimal mass ratio required for charge balancing the two electrodes was estimated using equation (2):


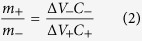


where *C*_*−*_ and *C*_*+*_ are the capacitances (in F g^−1^) measured at the same scan rate, using the three electrode system, for negative and positive electrodes, respectively while Δ*V*_*+*_ and Δ*V*_*−*_ denote the working potential window for the positive and negative electrodes, respectively. The required mass ratio for positive and negative electrode material (*m*_+_/*m*_*−*_) was thus estimated as 1.3 at 5 mV s^−1^.

### Electrochemical performance of asymmetric devices

ASCs were fabricated using the MWS and MWV electrodes (with m_+_ = 1.3 mg and m_−_ = 1 mg), Whatman glass fiber paper separator (pre-soaked in electrolyte) and 0.5 mol L^−1^ Li_2_SO_4_ aqueous electrolyte. The CV curves for the device were recorded in different voltage ranges but at a fixed scan rate of 50 mV s^−1^. The observed data are shown in Fig. 5d. The ASC exhibited quasi rectangular-shaped CV curves up to 1.8 V with redox peaks but no signature of H_2_/O_2_ evolution. However, in 1.9 V voltage window, there was a sharp rise in the current which indicated the release of H_2_ or O_2_ gases. Using the optimization experiments, stable operating voltage window for our fabricated ASC was inferred as 1.8 V. Although, high hydration energy of the used neutral electrolyte predicts ~1.6–2.2 V for water electrolysis into gases, the operating voltage window was limited to 1.8 V in the present case. This could be explained by taking into account the difference between the work functions of the used metal oxides. More detailed discussion is given later.

**Figure 6:**
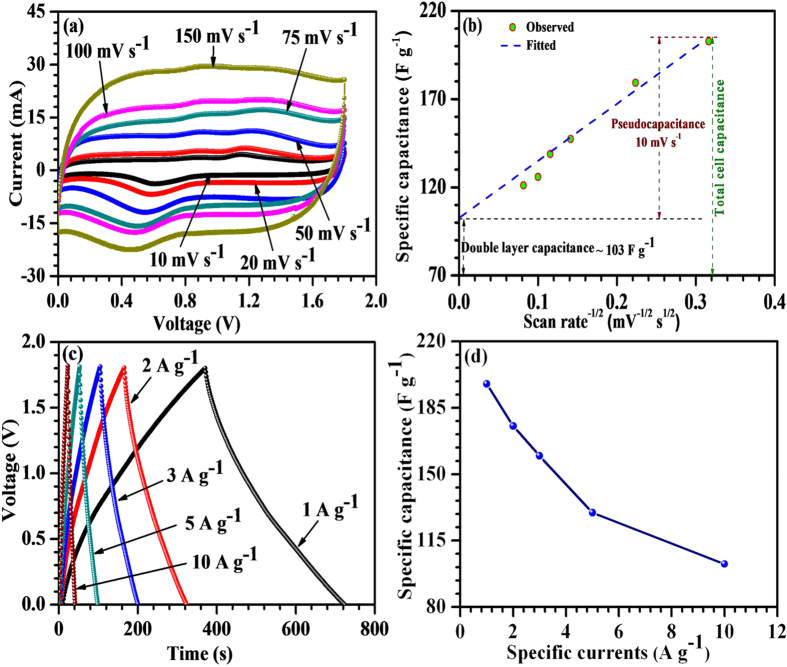
(**a**) Two electrode CV curves; (**b**) quantification of double-layer and pseudocapacitance; (**c**) galvanostatic charge-discharge curves; (**d**) rate capability for ASCs.

Figure 6a shows a series of CV measurements performed within 0–1.8 V at 10, 20, 50, 75, 100 and 150 mV s^−1^ scan rates for ASCs. Nearly rectangular-shaped CVs were observed at each scan rate with redox peaks. These peaks were more pronounced at small scan rates, which indicated convoluted contribution from double layer capacitance as well as pseudocapacitance. The nearly horizontal nature of these CVs indicates a small contribution from the equivalent series resistance of the ASCs^33^. The contribution from the electrode surface in ASCs operated at 10 mV s^−1^ was found to be ~50%, as shown in Fig. 6b. The contribution from the bulk of the electrodes decreased at higher scan rates indicating underutilized inner bulk of the active materials. The observed galvanostatic charge-discharge curves at specific currents of 1, 2, 3, 5 and 10 A g^−1^ in the voltage range 0–1.8 V are shown in Fig. 6c. Nearly symmetrical triangular-shaped charge/discharged curves confirmed the capacitive behavior. The non-linearity in the discharge curves reaffirmed the presence of pseudocapacitance as well as double layer capacitance in the convoluted cell capacitance. The specific capacitance values at 1, 2, 3, 5 and 10 A g^−1^ were found to be ~198, 175, 160, 130 and 103 F g^−1^, respectively. These values were calculated using the equation (3):


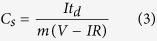


where *C*_*s*_ is the total cell capacitance (in F g^−1^), *I* is the specific current, *m* is the total mass of both the active materials excluding binder (i.e., *m*_+_ + *m*_*−*_ = 2.3 mg), *t*_*d*_ is the discharge time, *V* represents the stable operating voltage window and ‘*IR*’ is the measure of ohmic drop. It can be seen from Fig. 6d that ASCs demonstrated good rate capability with ~52% capacitance retention at 10 A g^−1^. The specific energy and power are estimated using the equations (4) and (5):


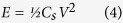



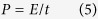


where *E, C*_*s*_*, V, P* and *t* stands for specific energy (mass normalized energy), specific capacitance, discharging voltage window excluding ohmic drop, specific power released and the discharge time, respectively.

The maximum specific energy obtained for the fabricated ASCs was ~89 Wh kg^−1^ at a specific power of ~903 W kg^−1^. The ASCs retain specific energy of ~46 Wh kg^−1^ at a specific current of 10 A g^−1^ while specific power reaches to ~9,002 W kg^−1^.

**Figure 7:**
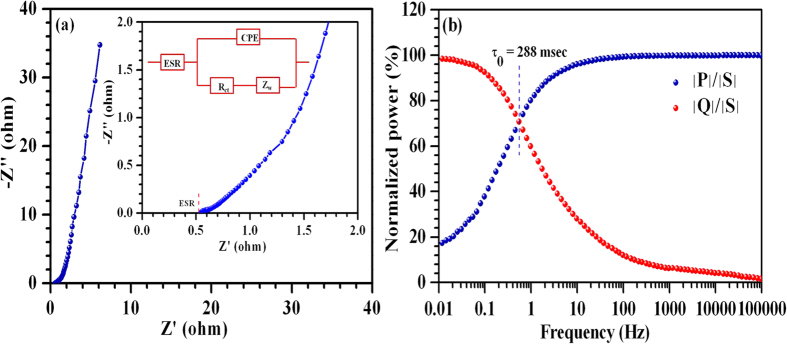
(**a**) Typical Nyquist plot and an equivalent circuit; (**b**) complex power analysis for ASCs.

Figure 7a shows the typical Nyquist plot observed for the fabricated ASCs in 1 mHz-100 kHz frequency range using a small ac perturbation of amplitude 5 mV. This plot can be subdivided into low and high frequency regions. The vertical rise in impedance value, at low frequencies, indicated good capacitive behavior. The region at 45°, in the moderate frequency region, is attributed to Warburg impedance arising due to frequency dependence of electrolyte ion diffusion inside the pores. The high frequency region possesses a small semicircle as manifested in the inset. This is indicative of the small charge transfer resistance at the electrolyte/electrode interfaces. The observed Nyquist plot can be represented by an equivalent circuit as shown in the inset of Fig. 7a. ESR is the equivalent series resistance comprising resistance of the electrode materials, electrolytes, current collectors and contact resistance (~0.55 Ω for the present case), CPE is the constant phase element, R_ct_ is the charge transfer resistance and Z_w_ is the Warburg impedance.

**Figure 8:**
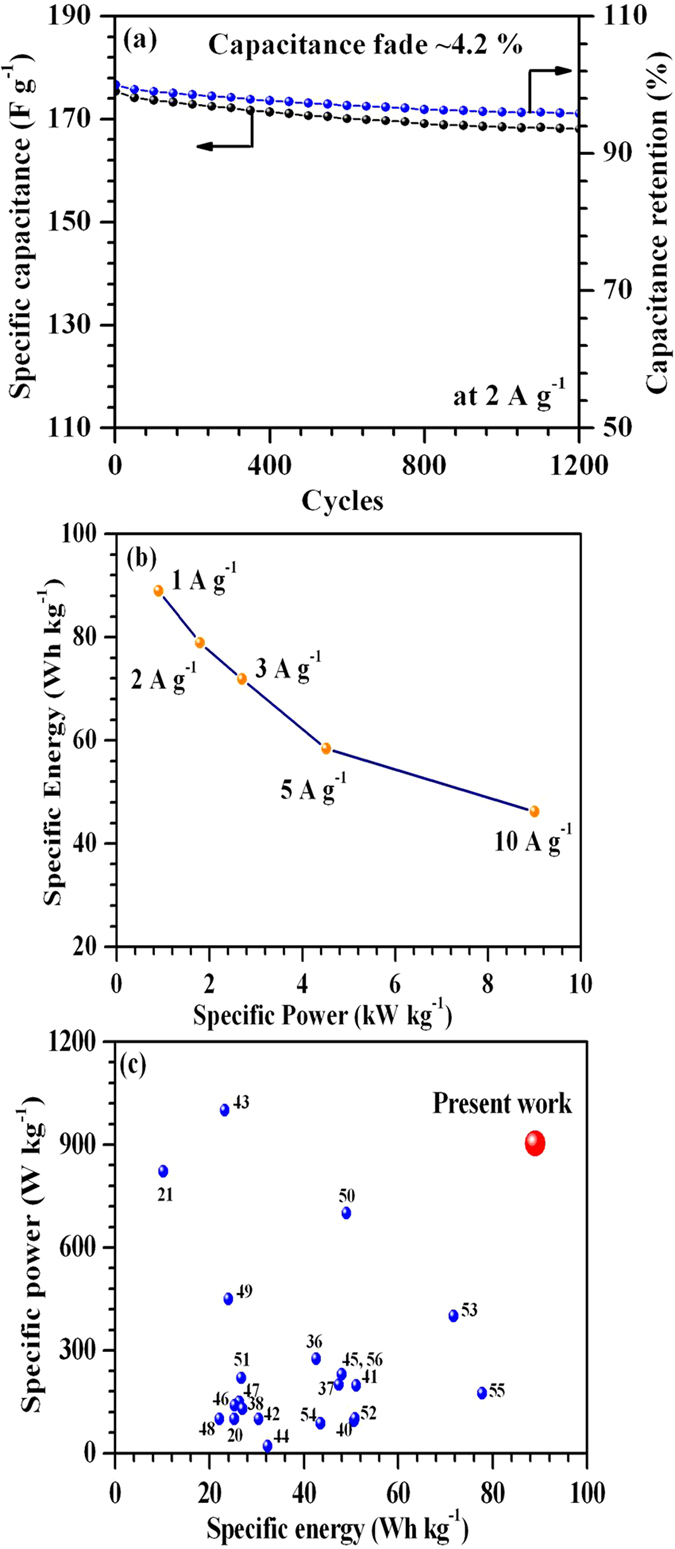
(**a**) cycling stability; (**b**) Ragone plot; (**c**) performance comparison for the fabricated ASCs.

Another figure of merit for the supercapacitors is the relaxation time constant (*τ*_*0*_), which can be estimated by analyzing the power dissipated into the system. Figure 7b shows the variation of real and imaginary part of the normalized complex power *S (ω)* as a function of frequency, which were calculated using equations (6, 7, 8):


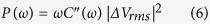



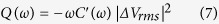



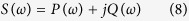


where 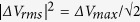 (*V*_*max*_ is the maximum amplitude of the applied ac perturbation) and *j* is imaginary number while the angular frequency *ω* is equal to 2πf. The *C’* and *C”* represent the real and imaginary part of the complex capacitance and calculated using following relations (equation (9) and (10)):


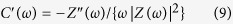



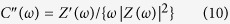


where *Z′* and *Z″* represents real and imaginary parts of the complex impedance *Z*. The normalized powers corresponding to phase angle of 45° converge at a frequency f_0_ ~552 mHz (known as relaxation frequency). Below this frequency, capacitive behavior supersedes the resistive behavior before reaching a pure capacitive characteristic at low frequency ~10 mHz. The relaxation time constant τ_0_ (equal to 1/2πf_0_) for the as-fabricated ASCs was found to be ~0.3 s. This clearly indicated fast charging-discharging capability of the fabricated ASCs. Cyclic stability is also essential for industrial applications of ASCs. Therefore, the fabricated ASCs were cycled at 2 A g^−1^. The data indicated only 4.2% capacitance fade after 1200 cycles (see Fig. 8a). The presence of oxygen vacancies can lead to an improved intercalation capacity and cyclic stability of V_2_O_5_ structures and ultimately, to an improved cyclic stability of ASCs^34^,^35^.

The Ragone plot (specific energy versus power plot) for fabricated ASCs at different specific currents is given in Fig. 8b. The obtained specific energy and power values were compared with those reported previously for ASCs based on aqueous electrolyte. This is graphically shown in Fig. 8c^20^,^21^,^36^,^37^,^38^,^39^,^40^,^41^,^42^,^43^,^44^,^45^,^46^,^47^,^48^,^49^,^50^,^51^,^52^,^53^,^54^,^55^,^56^. It is clear from Fig. 8c that our ASCs exhibit much superior specific energy whilst maintaining high specific power. The above results establishes the relevance of using these metal oxides/MW composites for fabricating high-performance ASCs in aqueous electrolytes. A more detailed comparison with structures of the fabricated ASCs, used electrolyte, operating voltage window and cyclic stability is also given in supplementary Table S1.

## Discussion

**Figure 9:**
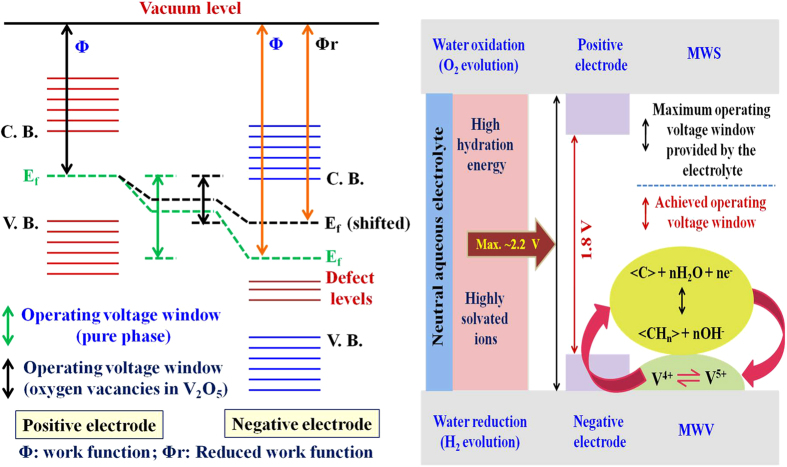
Energy band diagram explaining the stable and expanded operating voltage window achieved in the present ASCs.

The electrode potential can be defined against a reference electrode (known as electrochemical scale) or with respect to electron energy at rest in vacuum (known as physical scale)^57^,^58^,^59^. When it is defined on a physical scale, the working voltage window of an electrochemical cell is given by:


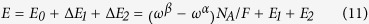


where *ω*^*α*^ and *ω*^*β*^ represents the work functions while *ΔE*_*1*_ and *ΔE*_*2*_ denotes the potentials of positive and negative electrodes, respectively and *N*_*A*_ stands for Avogadro’s constant^36^. In an ASC, the difference in the work function of the two metal oxides widens the operating voltage window beyond the decomposition energy of electrolyte because *ω*^*β*^−*ω*^*α*^ ≠ 0 and *E*_*1*_ ≈ −*E*_*2*_ for correctly charge balanced electrodes. For the present case, work function difference between SnO_2_ and V_2_O_5_ was ~2.35 eV, similar to that reported elsewhere^36^. Therefore, the expected stable operating voltage window was 2.2 V. In the present case, the observed voltage window of 1.8 V can be explained by the presence of oxygen vacancies in the V_2_O_5_ structures. Vanadium (V) oxide is a wide band gap oxide (d^0^ oxide) which has a tendency to form oxygen vacancies. The formation of the O-vacancies gives rise to other phases (VO_2_ for the present case) in addition to the V_2_O_5_ phase. This inference is supported by the XRD and XPS results for the MWV composite. The presence of the other VO_2_ phase changes d^0^-state of V_2_O_5_ to d^1^-state and ultimately results in a reduced work function^60^. Thus, V^5+^/V^4+^ phase with reduced work function forces water reduction at lower potentials for production and adsorption of atomic/nascent hydrogen on the MW surface. This results in a lower operating voltage than the predicted maximum achievable window (2.2 V). The modification in the band structure is schematically explained in Fig. 9.

## Conclusions

The synergistic effect between the pseudocapacitive nanostructures of SnO_2_ and V_2_O_5_ and carbon nanotubes lead to pronounced increase in the specific energy and power values delivered by the asymmetric supercapacitors. The fabricated asymmetric supercapacitors show superior electrochemical performance in comparison to those reported previously for ASCs in aqueous electrolytes. The charge balanced device returns the specific capacitance of ~198 F g^−1^ with corresponding specific energy of ~89 Wh kg^−1^ at 1 A g^−1^. Such high values can be attributed to: (a) high specific surface areas of the MWS and MWV along with good porosity that induce large interfacial contact region between the electrode material and electrolyte, (b) the positive synergistic effect between MW and the used metal oxides (c) the use of a neutral aqueous electrolyte with highly solvated ions and (d) a large difference in the work function of the used metal oxides.

## Methods

### Materials used

Multiwall carbon nanotubes (MWCNTs) (ID 3–5 nm; OD 20–25 nm; length 20 μm and purity 95.0%) were purchased from Nanocyl (Belgium). Tin chloride (SnCl_4_.2H_2_O) and ammonium metavanadate (NH_4_VO_3_) were purchased from Loba chemicals Pvt. Ltd., India. Hydrochloric acid (HCl; 38%), isopropanol and ammonium hydroxide (25% ammonia solution) were purchased from Loba Chemie Pvt. Ltd., India. All the chemicals were used without further purification. Whatman glass microfiber filters (GF/C^TM^; diameter 47 mm) were purchased from GE Healthcare UK Limited, UK.

### Material synthesis and characterizations

Initially, functionalization of the MWCNTs (MW) was carried out by refluxing them in concentrated HNO_3_ (69%) at 120 °C for 12 h. For the MWS composite, 200 mg functionalized MW were added in 300 ml de-ionized (DI) water and stirred overnight at room temperature to get homogenous dispersion. The solution of tin chloride (0.2 g SnCl_4_.2H_2_O in 100 ml of DI water) with 3 ml of 38% HCl was then added to MW dispersion and heated at 90 °C for 12 h in an oil bath. Finally, product was collected by filtration followed by washing several times with DI water and finally dried overnight at 70 °C in a vacuum oven. To synthesize MWV composite, first, ammonium vanadate/MW was prepared by refluxing 200 mg functionalized MW and 25 g of V_2_O_5_ in 187 ml isopropanol and 35 ml of 4.2 M NH_4_OH aqueous solution at 70 °C for overnight. The product was obtained by filtration followed by washing several times with DI water and drying at 60 °C for 36 h in a vacuum oven. MWV was obtained by heating the product thus obtained at 500 °C for 10 h in N_2_ environment. Pure SnO_2_ and V_2_O_5_ were also synthesized by following the same procedures.

Powder X-ray diffraction (XRD) patterns were collected for the synthesized materials using PAN Analytical diffractometer with Cu-Kα radiation at wavelength 1.5406 Å in 2θ range 15–70°. For morphological study, the samples were subjected to field emission scanning electron microscopy (SEM CARL ZEISS SUPRA 40) and high resolution transmission electron microscopy (TEMFEI-TECHNAI G220S-Twin operated at 200 kV). X-ray photoelectron spectroscopy (XPS) measurements were carried out with the help of PHI 5000VERSAProbe II X-ray photoelectron spectrometer having Al-Kα as incident photon energy. Thermogravimetric analysis (TGA) was performed in O_2_ environment at 10 °C min^−1^ using NETZSCH STA 409 PC/PG thermal analyzer. Fourier transform infrared spectroscopy (FTIR) spectra were collected using Spectrum BX FTIR (Perkin Elmer version 5.3). The Brunauer-Emmett-Teller (BET) surface area and porosity were measured by analysing adsorption-desorption isotherms obtained from Micromeritics Gemini V Model 2365 and Gemini VII Model 2390t.

### Device fabrication and electrochemical characterizations

To prepare electrodes, slurry was prepared using 95% of the active materials (i.e., MW, MWS, SnO_2_, V_2_O_5_ or MWV) and 5% polyvinylidene fluoride (PVDF) in 50 ml acetone and heated at 100 °C to get homogeneous and stable slurry. Then, slurry was drop casted on graphite sheet (100 μm thick; area ~1 cm^2^) to get desire mass loading. Finally, electrodes were dried at 100 °C before their use in device. Three electrode CV measurements were performed using a set-up provided by Metrohm Autolab comprising a reference electrode (Ag/AgCl; saturated KCl) and a counter electrode (Pt electrode). The mass of each active material used in three electrode CV measurements was ~1 mg. For the 3-electrode CVs of SnO_2_, the starting potential is -0.1 V (*vs.* Ag/AgCl) and starting step of the CV measurement is positive sweep. However, in case of V_2_O_5_, the starting potential is −0.1 V (*vs.* Ag/AgCl) but the starting step of the CV measurement is negative sweep. The ASCs were fabricated by sandwiching a glass fibre paper (soaked in 0.5 M Li_2_SO_4_ aq. electrolyte) between two electrodes with required mass ratio (m_+_/m_*−*_ = 1.3 with m_+_ = 1.3 mg and m_*−*_ = 1.0 mg) as derived from charge balanced equation (2). The whole device is slightly pressed between two stainless-steel clamps. The electrochemical characterizations of ASCs were performed using a Metrohm Autolab (Galvanostat/Potentiostat) associated with Nova 1.10.1.9 software.

## Additional Information

**How to cite this article**: Singh, A. and Chandra, A. Significant Performance Enhancement in Asymmetric Supercapacitors based on Metal Oxides, Carbon nanotubes and Neutral Aqueous Electrolyte. *Sci. Rep.* **5**, 15551; doi: 10.1038/srep15551 (2015).

## Supplementary Material

Supplementary Information
