# Supplementary material for: Significant Performance Enhancement in Asymmetric Supercapacitors based on Metal Oxides, Carbon nanotubes and Neutral Aqueous Electrolyte
Source: Sci Rep. 2015 Oct 23;5:15551. doi: 10.1038/srep15551 (PMC4616038; doi:10.1038/srep15551)
Supplement: Supplementary Information [file srep15551-s1.pdf]

Supplementary Information

Significant Performance Enhancement in Asymmetric Supercapacitors based on Metal Oxides, Carbon nanotubes and Neutral Aqueous Electrolyte

*By Arvinder Singh, Amreesh Chandra**

[*] Dr. A. Chandra

Department of Physics,

Indian Institute of Technology Kharagpur, Kharagpur-721302, West Bengal, India

E-mail: achandra@phy.iitkgp.ernet.in

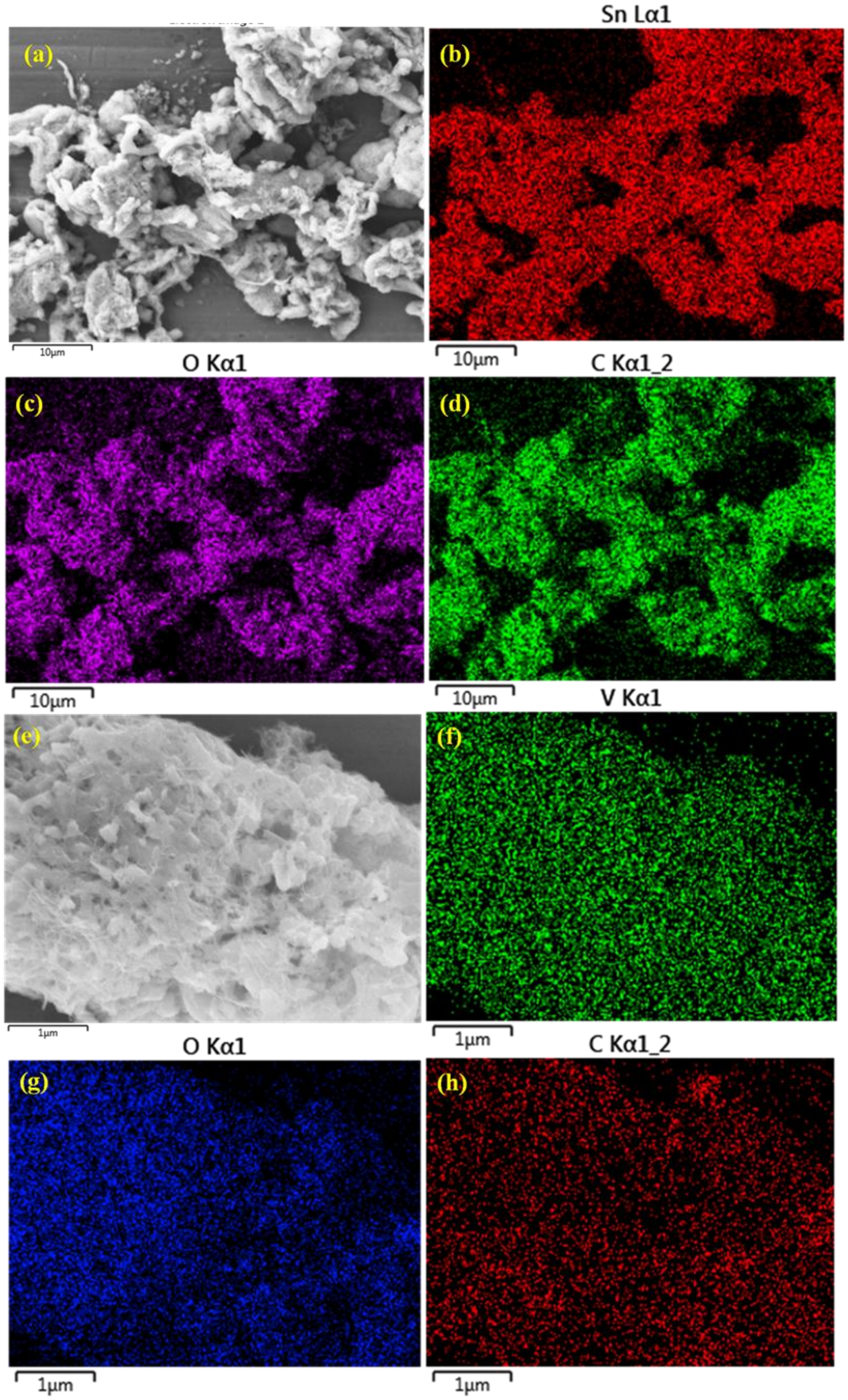

Fig. S1. Focused ion beam (FIB) elemental maps for the awaited elements in (a-d) MWS; (e-h) MWV composite materials each scanned for 5 minutes

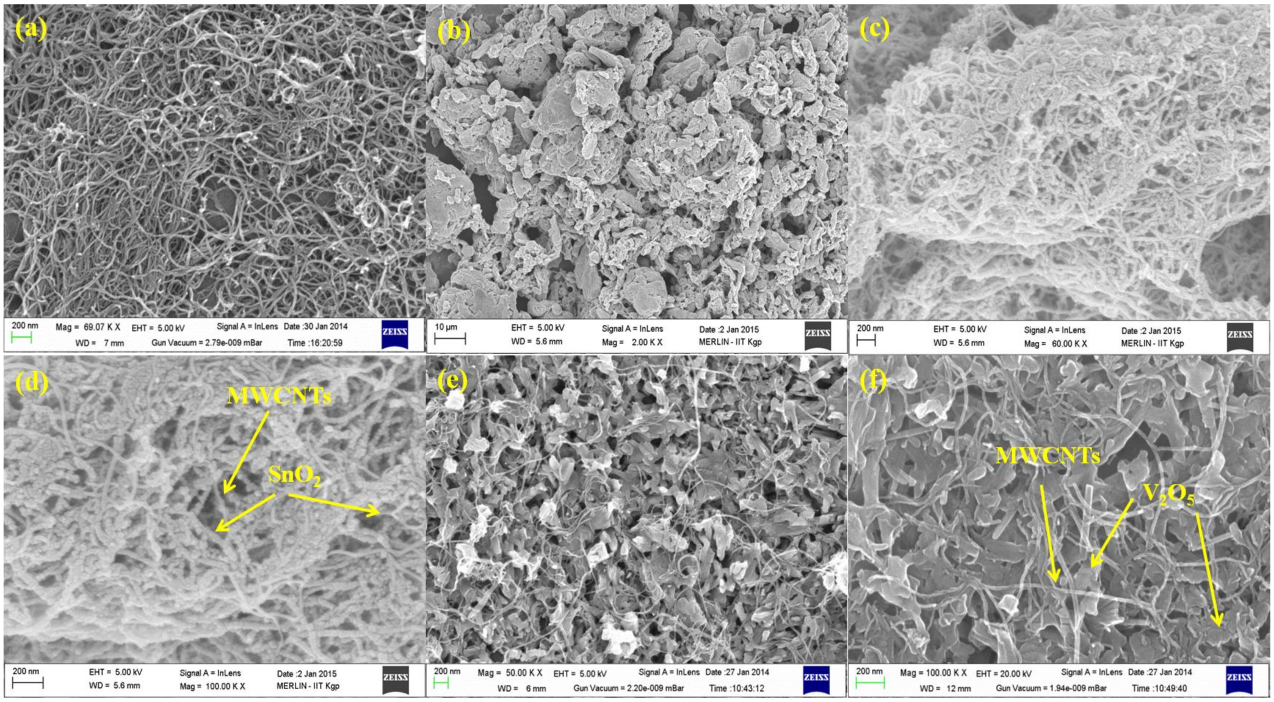

Fig. S2. FESEM micrographs observed for (a) MWCNTs (MW); (b-d) MWS; (e-f) MWV samples

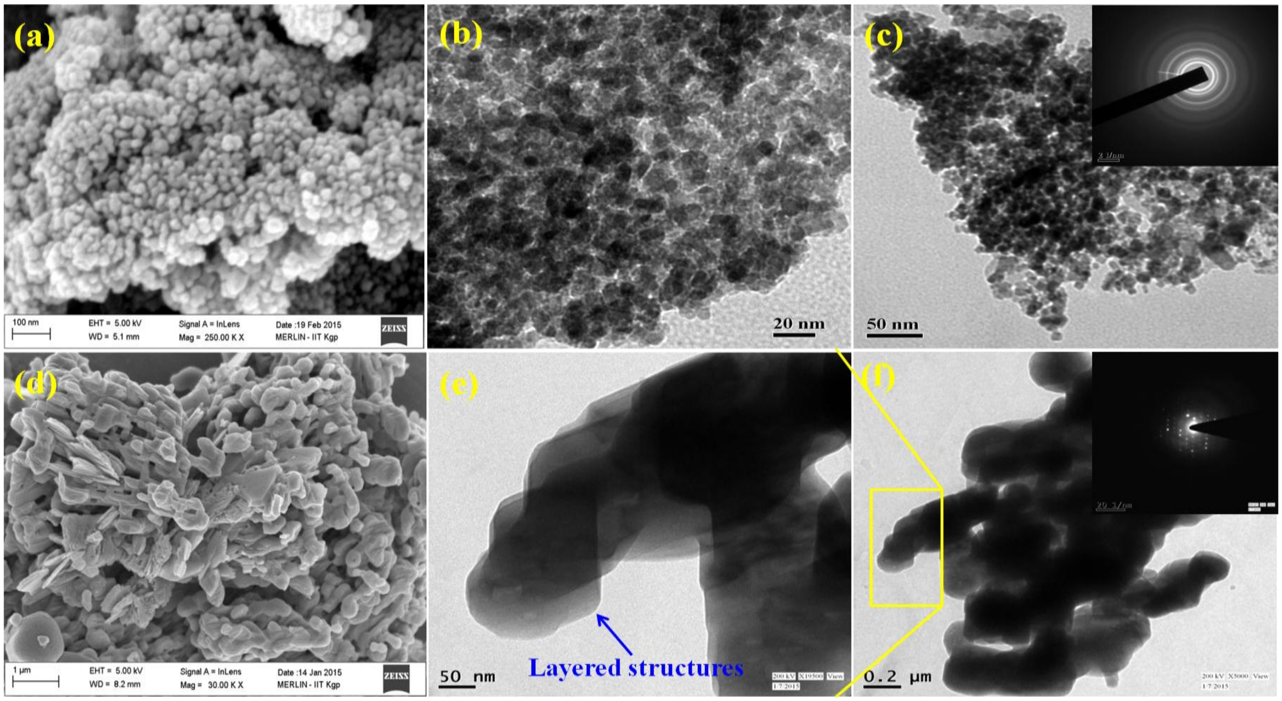

Fig. S3. FESEM and TEM micrographs for (a-c) SnO_2; (d-f) V_2O_5 samples. Inset to (c) and (f) are their respective SAED patterns.

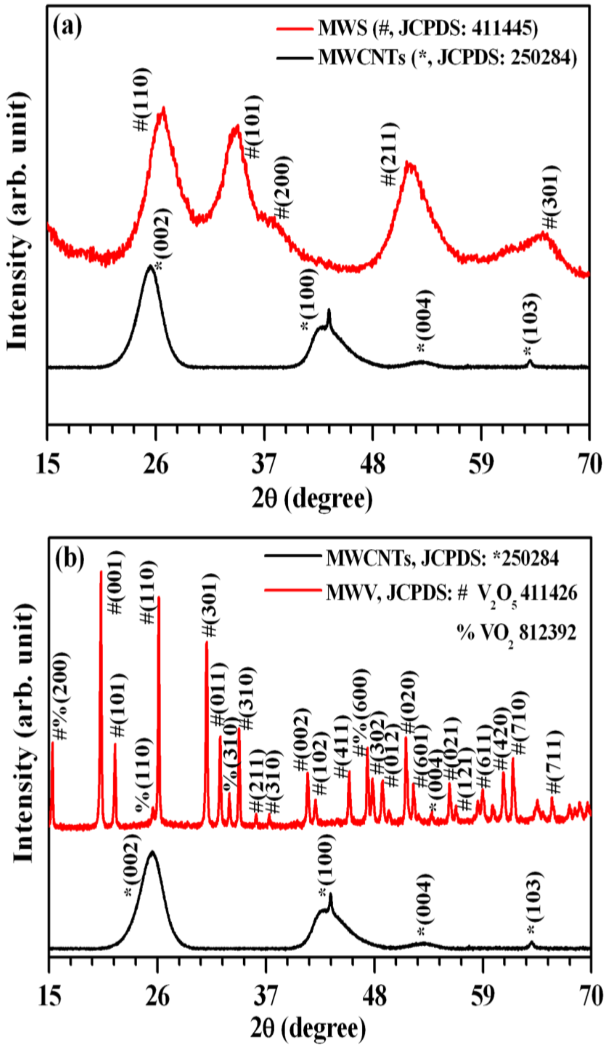

Fig. S4. Typical XRD patterns observed for (a) MW and MWS; (b) MW and MWV composite materials

In order to investigate the crystal structure and phase formation, powder XRD patterns were collected for MWCNTs, MWS and MWV composites (Fig. S4). In Fig. S4a, the XRD pattern of MW showed characteristic graphitic peaks at 2θ ~25.4° and ~43.5° corresponding to (002) and (100) planes, respectively. These two characteristics peaks have nearly vanished in the MWS composite whereas newly originated peaks could be readily indexed to tetragonal phase of SnO_2 according to Joint Committee on Power Diffraction Standards (JCPDS) card no 411445. The disappearance of the characteristics peaks for MW in MWS composite could be assigned to covering of MWCNTs surface with SnO_2 nanoparticles as evident from HRTEM micrographs. Moreover, In the case of MWV composites, all the peaks excluding those for MWCNTs could be indexed to V_2O_5/VO_2 phase according to JCPDS files no. 411426/812392 as shown in Figure S4b. The vanishing of the characteristics peaks of MWCNTs is due to dispersion of CNTs underneath the layered structures of V_2O_5/VO_2, which is in well agreement with HRTEM results.

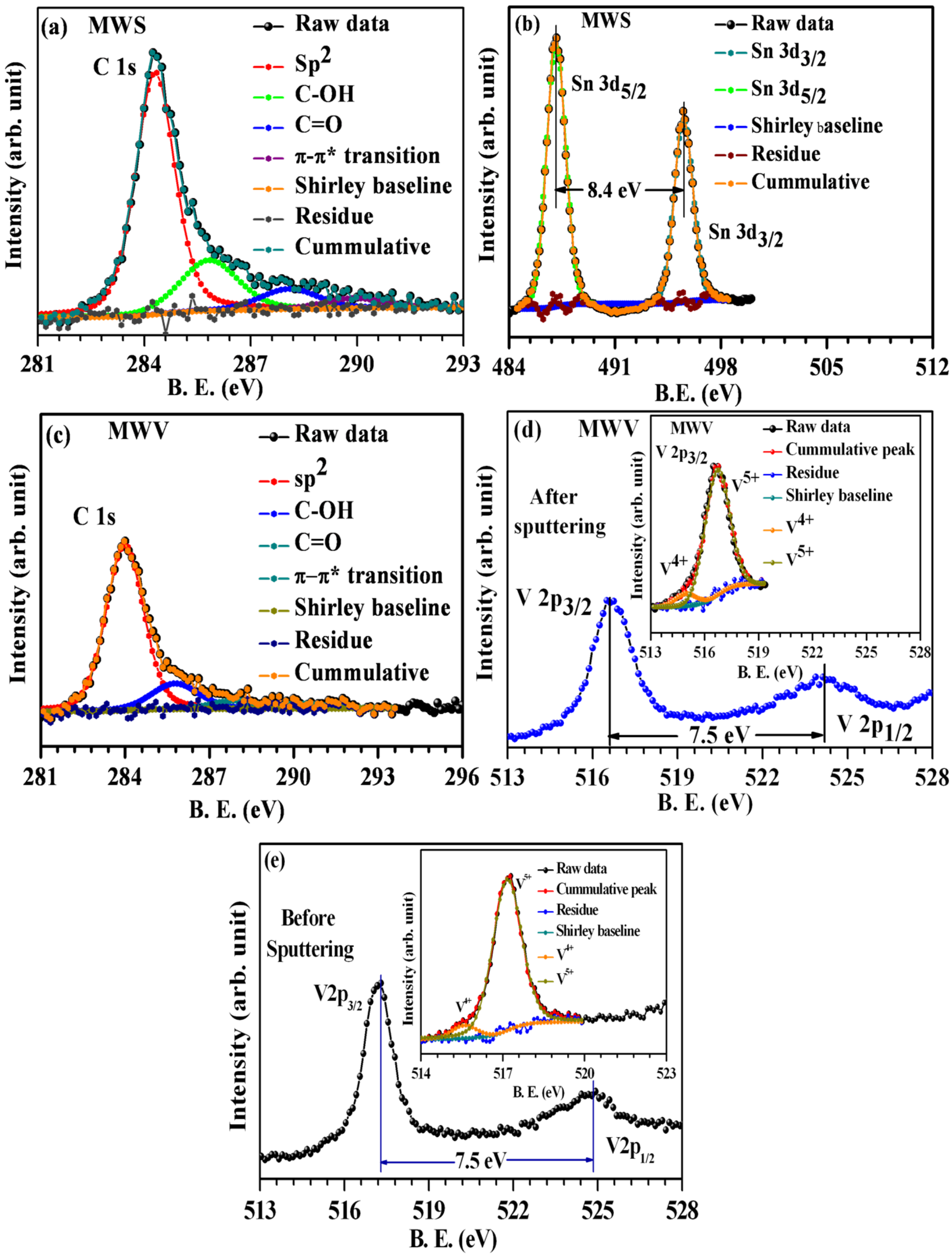

Fig. S5. XPS spectra observed for (a) C 1s for MWS; (b) Sn 3d for MWS; (c) C 1s for MWV; (d) V 2p for MWV composite after sputtering; (e) V 2p for MWV composite before sputtering. An inset to (d) and (e) shows de-convoluted V 2p$_{3/2}$ peak.

To determine the chemical composition and valance state of the present elements, the core level X-ray photoelectron spectra (XPS) were recorded for the MWS and MWV composites. The XPS spectrum of standard Ag sample was used to remove surface charging effect. The sample's surface was sputtered up to few nanometers before recording of the XPS spectra. Figure S5a-b shows C 1s and Sn 3d core level spectra for MWS composite, respectively. The C 1s peak is appeared at 284.2 eV with several deconvoluted peaks underneath the C 1s peak, which could be assigned to C=C Sp^2 carbons (284.3 eV), C-OH (285.9 eV), C=O (288.2 eV) and π-π* transition (290.2 eV) in the MW structures, respectively. Moreover, the observed two symmetrical peaks (i.e., Sn $3d_{5/2}$ at ~487.1 eV and Sn $3d_{3/2}$ at ~495.5 eV) with a binding energy separation of 8.4 eV could be assigned to presence of the SnO_2 in the MWS composite (Fig. S5b). The estimated atomic percentage from XPS spectra for the elements present in MWS composite is: C=41.1 %, O=39.3 % and Sn=19.6 % suggesting single phase of SnO_2 (as O and Sn atomic percentage ratio is ~2), which is in well agreement with XRD results. Figure S5c-d shows XPS core level spectra of C 1s and V 2p for the MWV composite, respectively. The C 1s is originated at 284.0 eV; possessing several deconvoluted peaks at 284.0 eV, 285.8 eV, 287.8 eV and 290.4 eV, which could be attributed to sp^2 carbons, C-OH, C=O and π-π* transition (refurbishment of aromaticity after composite synthesis) in MW structure, respectively. Furthermore, in V 2p spectra (before sputtering Fig. S5e and after sputtering Fig. S5d), two characteristic peaks (i.e., V $2p_{3/2}$ and V $2p_{1/2}$) are appeared at ~516.7 eV (before sputtering ~517.3 eV) and ~524.2 eV (before sputtering ~524.8 eV), respectively with an energy separation of ~7.5 eV. An inset to Fig. S5d-e shows de-convolution of the V $2p_{3/2}$ peak into two components; high energy component peak is assigned to V^{5+} and low energy component peak (smaller) is attributed to presence of V^{4+} in V_2O_5 structures. The presence of V^{4+} state is found to be increased from ~9.4% (before sputtering) to ~16.8% (after sputtering) due to sputtering of the sample surface. This result shows

the presence of small fraction of VO_2 phase in assistance with V_2O_5 phase (before sputtering) and is in well agreement with the XRD results. The atomic percentage of the elements presents in MWV composite is: C=42.3 %, O=41.4 % and V=16.2 %.

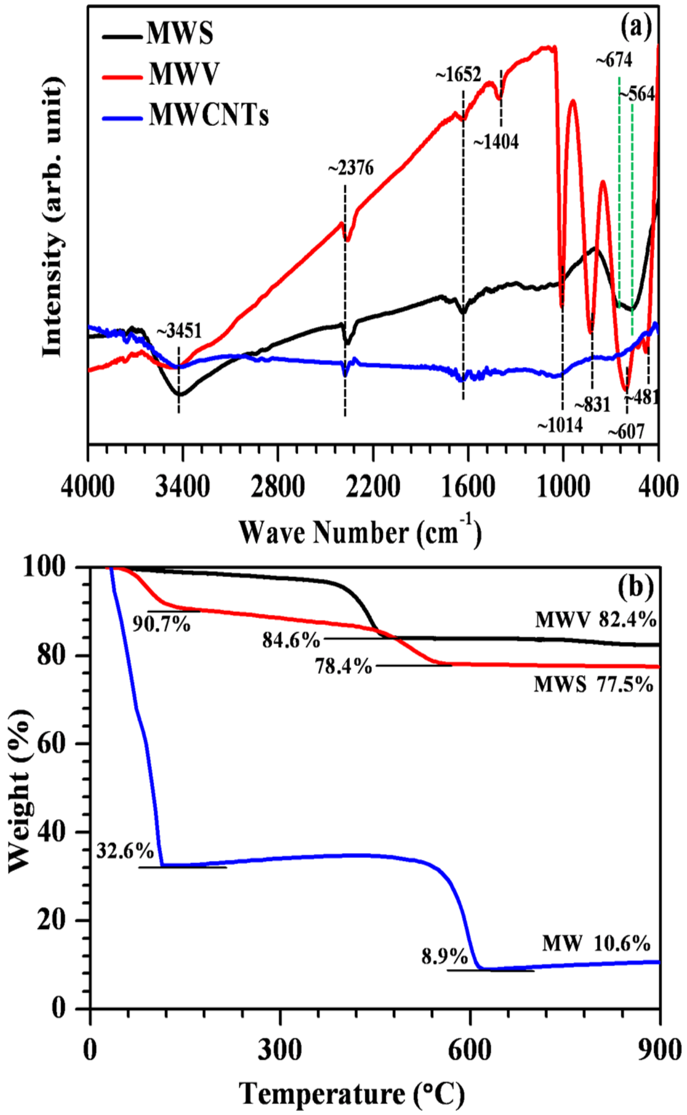

Fig. S6. (a) FTIR spectra; (b) TGA curves for the acid treated MW, MWS and MWV samples

Fourier transfer infrared spectroscopy (FTIR) was performed on MW, MWS and MWV samples which are given in Fig. S6a. Due to the presence of MW, the observed FTIR spectra for all the samples are exhibiting various peaks at ~3451 cm^{-1}, ~2376 cm^{-1}, ~1652 cm^{-1} and ~1404

cm^{-1}. These peaks are arising due to O-H stretching vibrations from carboxyl groups (O=C−OH and C−OH), O−H stretching from strongly hydrogen-bonded −COOH groups, skeletal vibrations of unoxidized C=C carbons (MWCNTs backbone) and O-H bending deformation vibration in carboxylic group, respectively. Two newly originated peaks for the MWS composite at ~674 cm^{-1} and ~564 cm^{-1} are attributed to stretching vibrations of Sn-O bonds, which confirms the presence of SnO_2 in the MWS matrix. The FTIR spectra of the MWV is possessing several additional absorption peaks appearing at ~1014 cm^{-1} and ~831 cm^{-1}, which are assigned to (vanadium)-O (vanadyl oxygen) stretching mode and anti-symmetric stretching vibration of the V-O (bridging oxygen)-V group. Two peaks located at ~607 cm^{-1} and ~481 cm^{-1} are due to edge shearing 3V-O stretching and the bridging V-O (bridging)-V deformations in V_2O_5 structures of the MWV composite. These FTIR results reveal the formation of the MWS and MWV composites.

To evaluate the weight percentage of SnO_2 and V_2O_5 in the MWS and MWV composites more precisely, thermogravimetric analysis (TGA) was performed for the functionalized MW and as-prepared MWS and MWV composites (Fig. S6b). The removal of water gives first weight loss below 100 ºC in the MWS composite and MW samples. Further weight loss in all the samples (500-600 ºC) is due to the decomposition of the functionalized MW. The weight percentages of SnO_2 and V_2O_5 in MWS and MWV composites are found to be 66.9 wt% and 71.8 wt%, respectively; estimated by comparing residual weight percentage of the functionalized MW (10.6 wt%).

Table S1: Performance comparison of our ASCs with previously reported aqueous ASCs

ASCs structure	Electrolyte used	Operating Voltage [V]	Specific energy [Wh kg^{-1}]	Specific power [W kg^{-1}]	Cycles and capacitance fade	Reference no
MnO_2//graphene	Na_2SO_4	2.0	25.2	100	500; 4%	20
Bi_2O_3//Activated carbon	Li_2SO_4	1.6	10.2	822.6	-	21
Graphene/MnO_2//Graphene/MoO_3	Na_2SO_4	2.0	42.6	276	1000; -	36
MnO_2//Mesoporous CNTs	Na_2SO_4	2.0	47.4	200	1000; 10%	37
Graphene/MnO_2/fFWCNTs// activated carbon/fFWCNTs	Na_2SO_4	2.0	27	130	2000; ~5%	38
CNTs/MnO_2//CNTs/In_2O_3	Na_2SO_4	2.0	25.0	-	-	39
$Ni(OH)_2$/CNT/Ni foam//Activated carbon	KOH	1.8	50.6	95	3000; 17%	40
Graphene/MnO_2//Activated carbon nanofiber	Na_2SO_4	1.8	51.1	198	1000; 3%	41
Graphene/MnO_2//Graphene	Na_2SO_4	2.0	30.4	100	1000, 21%	42
MnO_2//Graphene hydrogel	Na_2SO_4	2.0	23.2	1000	5000; 16.6%	43
Activated graphene/MnO_2// Activated graphene	Na_2SO_4	2.0	32.3	21	5000; 19.5%	44
Graphene/$Ni(OH)_2$//Graphene/RuO_2	KOH	1.5	48.0	230	5000; 8%	45
$K_{0.27}MnO_2$//Activated carbon	K_2SO_4	1.8	25.3	140	10000; >2 %	46
Graphene/Polyaniline//Graphene/ RuO_2	KOH	1.4	26.3	150	2500; 30%	47
Carbon spheres/MnO_2//Carbon spheres	Na_2SO_4	2.0	22.1	100	1000, 1%	48
MnO_2//FeOOH	Li_2SO_4	1.85	24.0	450	2000; 15%	49
MWCNTs/NiS//Graphene	KOH	1.4	49.0	700	1000, 8%	50
V_2O_5 nanofiber//Polyaniline nanofiber	KCl	2.0	26.7	220	2000, 27%	51
3D Porous graphene/MnO_2// Graphene/Ag	Na_2SO_4	1.8	50.8	101.5	-	52
Ni-Co oxide//Activated polyaniline derived carbon	KOH	1.6	71.7	400	4000; ~12%	53
CoO/Polypyrrole//Activated carbon	NaOH	1.8	43.5	87.5	20000; 8.5%	54
$Ni(OH)_2$/Graphene//Graphene	KOH	1.6	77.8	174.7	3000; 5.7%	55
$NiCo_2O_4$/Graphene//Functionalized Activated carbon	KOH	1.55	48	230	5000; <2%	56
**SnO_2/MWCNT//V_2O_5/MWCNT**	**Li_2SO_4**	**1.8**	**89.0**	**903.3**	**1200; 4.2%**	**Present**
